# Diagnostic Fluidity: Inter- and Intra-Disease Considerations

**DOI:** 10.1192/j.eurpsy.2022.117

**Published:** 2022-09-01

**Authors:** R. Krueger

**Affiliations:** University of Minnesota, Psychology, Minneapolis, United States of America

**Keywords:** Nosology, Classification, Psychopathology, Comorbidity

## Abstract

Traditionally, psychopathology has been classified based on the publications of authoritative bodies, such as the American Psychiatric Association’s Diagnostic and Statistical Manual of Mental Disorders (APA’s DSM). Recently, researchers have expressed an interest in basing classification more on data, as opposed to authority. This movement led to the formation of the Hierarchical Taxonomy of Psychopathology (HiTOP) consortium. Working from data, the HiTOP approach emphasizes dimensions of human individual differences that are arranged hierarchically, as opposed to categories that are arranged based on traditional DSM chapter rubrics. In this talk, I will describe the origins and status of the HiTOP approach, as well as current and future HiTOP directions and priorities.

**Disclosure:**

No significant relationships.

